# Case report of a young child with disseminated histoplasmosis and review of hyper immunoglobulin e syndrome (HIES)

**DOI:** 10.1186/1476-7961-9-14

**Published:** 2011-11-29

**Authors:** Wilson S Robinson, Sandra R Arnold, Christie F Michael, John D Vickery, Robert A Schoumacher, Eniko K Pivnick, Jewell C Ward, Vijaya Nagabhushanam, Dukhee B Lew

**Affiliations:** 1 Children's Foundation Research Center at Le Bonheur Children's Hospital, University of Tennessee Health Center, Memphis, TN 38103-2800, USA; 2 Department of Pediatrics, University of Tennessee Health Science Center, Memphis, TN 38103, USA; 3 Advanced Diagnostics Immunology Laboratories, National Jewish Hospital, Denver, CO 80015, USA

## Abstract

Type 1 hyper IgE syndrome (HIES), also known as Job's Syndrome, is an autosomal dominant disorder due to defects in STAT3 signaling and Th17 differentiation. Symptoms may present during infancy but diagnosis is often made in childhood or later. HIES is characterized by immunologic and non-immunologic findings such as recurrent sinopulmonary infections, recurrent skin infections, multiple fractures, atopic dermatitis and characteristic facies. These manifestations are accompanied by elevated IgE levels and reduced IL-17 producing CD3+CD4+ T cells. Diagnosis in young children can be challenging as symptoms accumulate over time along with confounding clinical dilemmas. A NIH clinical HIES scoring system was developed in 1999, and a more recent scoring system with fewer but more pathogonomonic clinical findings was reported in 2010. These scoring systems can be used as tools to help in grading the likelihood of HIES diagnosis. We report a young child ultimately presenting with disseminated histoplasmosis and a novel STAT3 variant in the SH2 domain.

## Introduction

Type 1 hyper IgE syndrome (HIES) is an autosomal dominant disorder due to defects in signal transducer and activator of transcription 3 (STAT3) signaling [1,2]. HIES was first described in 1966 and called Job's syndrome for the clinical manifestation of recurrent staphylococcal abscesses [3]. The association with elevated IgE levels was discovered in 1972 and was referred to as Buckley syndrome [4] which was found to be the same condition as Job's syndrome. The genetic mutation of HIES was discovered in 2007. Minegishi *et al*. and Holland *et al*. reported heterozygous dominant-negative mutations in STAT3 in the DNA-binding and SH2 domains [5,6]. STAT3 plays an important signaling role in Th17 differentiation.

Type 1 HIES is characterized by immunologic and non-immunologic findings. These manifestations are accompanied by elevated IgE levels and reduced IL-17 producing CD3+CD4+ T cells. Immunologic findings include newborn rash, recurrent sinopulmonary infections, recurrent skin infections, recurrent cyst-forming pneumonias, eczema, mucocutaneous fungal disease, eosinophilia and elevated IgE. Non-immunologic findings include characteristic face, retained primary teeth, multiple fractures, scoliosis, hyperextensibility, Chiari I malformations and craniosynostosis.

Diagnosis in young children can be challenging as symptoms accumulate over time along with confounding clinical dilemmas. A clinical HIES scoring system by the National Institutes of Health (NIH) was developed in 1999 [1]. A more recent scoring system with fewer but more pathogonomonic clinical findings was reported in 2010 [2]. These scoring systems can be used as tools to assess the likelihood of HIES diagnosis.

Also reported are autosomal recessive forms of HIES, i.e. defects in dedicator of cytokinesis 8 protein (DOCK8) and tyrosine kinase 2 (TYK2). These forms are less common, mostly described in consanguineous communities and have several different clinical distinctions.

## Case Report

This patient with wiry hair and square asymmetric face, was initially hospitalized at 5 months of age for pneumonia, multiple fractures, eczema with facial folliculitis but without cold abscess, wheezing, or milk allergy. Evaluation for non-accidental trauma was ongoing. Laboratory findings were as follows: IgE 187 IU/mL, IgA < 6.3 mg/dL, and eosinophil count 3,500/mm3. IgG, IgM, lymphocyte mitogen stimulation and cell surface markers were normal. Family history was noncontributory. His IgA normalized over the next 2 years. He had nine subsequent hospitalizations due to the following issues: rib fractures and pneumonia, respiratory distress secondary to subglottic cyst, gastroenteritis with dehydration, pneumonia requiring mechanical ventilation, severe dysphagia/aspiration pneumonia requiring G-Tube/Nissen fundoplication, and *Streptococcus pneumoniae *pneumonia with parapneumonic effusion. He also developed pneumatoceles and required left thoracotomy with bleb plication/pleurodesis. Other pertinent complications included recurrent ear infections and thrush, developmental delay, osteopenia, eczema, cellulitis, and allergic sensitization to egg, cat and dust mites. Further evaluation included normal chromosomes (46XY), CPK, vitamin D, calcium, PTH, and sweat chloride, along with negative HIV screening.

In July 2010, at 33 months of age, he presented with hepatosplenomegaly, hypoxia and respiratory distress. A diffuse opacity was seen on chest xray. Bronchoalveolar lavage showed extracellular and intracellular yeast suspicious for *Histoplasma capsulatum*. Disseminated histoplasmosis was confirmed by urine and serum antigen levels (both above measurable test limit).

**Figure 1:**
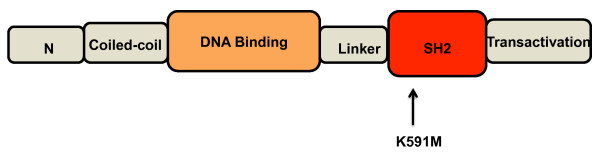
**STAT3 Novel Variant**. Our patient's mutation on a schematic primary structure of STAT3 gene. STAT3 analysis revealed a missense mutation in exon 20 within the SH2 domain [c.1772A > T, amino acid change p.Lys591Met].

Due to disseminated infection and the complexity of his clinical course, the question of primary immunodeficiency was revisited. His IgE level on initial presentation at 5 months of age was 187 IU/mL, and by 33 months of age had increased to 1,106 IU/mL. The clinical score for HIES progressed to 46 (Table 1). Dihydrorhodamine, Mannan-binding-lectin, CH50 and mitogen stimulation were normal. Pneumococcal titers were flat despite a history of *S. pneumoniae *infection and vaccination with 3 out of 4 Prevnar7 doses. Cell surface markers were found to be normal except for slightly elevated activated T cells at 12% and slightly decreased CD4+CD45+ RA-/RO+ memory T cells at 15% with normal reference range of 16-46%. Flow cytometric analysis of Th1 and Th17 cells [performed by the Advanced Diagnostic Immunology Laboratories National Jewish Health (Denver, CO) as a research development study with a minor modification (5 versus 6 hour stimulation) of the method of Ma *et al*.] [7] revealed low Th17 or IL-17 producing cells (0.3% of CD4+ T cells; date-matched normal control: 2.1%). Th17:Th1 ratio was also low (9:1,000). STAT3 gene analysis revealed a missense mutation in exon 20 within the Src-homology (SH2) domain [c.1772A > T, amino acid change p.Lys591Met] (Figure 1).

**Table 1:** Chronologic HIES scoring

Age (mo) Date	9 June, 2008	20 May, 2009	23 Aug, 2009	33 July, 2010
IgE score (IU/ml)	1 (187)	1 (371)	8 (1,011)	8 (1,106)
Skin abscess	0	0	0	2
Pneumonia	2	4	6	8
Lung anomalies	0	0	8	8
Fractures	4	4	4	8
Eczema	2	2	2	2
URIs/year	2	2	2	2
Candidiasis	1	1	1	1
Serious infections	0	0	0	4
Young age correction	7	5	5	3
**NIH Score**	**19**	**19**	**36**	**46**
Pneumonia	N/A	N/A	6 × 2.5	8 × 2.5
Pathologic fractures	N/A	N/A	4 × 3.33	8 × 3.33
**STAT3 Score**	**N/A**	**N/A**	**28.32**	**46.64**

NIH score: unlikely, < 20; indeterminate, 20-40; suggestive of AD-HIES, > 40

STAT3 score: possible, > 30+IgE ≥ 1,000 IU/ml, probable: > 30+IgE ≥ 1,000 IU/ml + low Th17 and/or positive F.Hx; definitive, > 30+IgE ≥ 1,000 IU/ml + heterozygous STAT3 mutation

Informed consent was obtained and approved by UTHSC IRB.

## Discussion

We report a case of disseminated histoplasmosis in a young child with STAT3-HIES [c.1772A > T, amino acid change p.Lys591Met]. Our patient's missense mutation in the SH2 domain has not been previously reported. Woellner *et al*. identified a missense mutation in the same amino acid [c.1771A > G, amino acid change p.Lys591Glu] but no clinical information is available [2].

### Genetics

AD-HIES is now defined by heterozygous mutation detection in STAT3 which contains 24 exons and 3 splice variants. STAT3 sequencing became commercially available in mid-2009 which provides the opportunity for more expedited and objective diagnosis but must be weighed by the level of clinical suspicion due to healthcare cost burden. Over 95% of AD-HIES have detectable STAT3 mutations and all domains have had mutations described including the N-terminal, coiled-coil, DNA-binding, linker[8], SH2 and transactivation domains.

Most of these mutations affect either the DNA-binding domain or the SH2 domain. Genotype-phenotype correlations involving most common domains and hotspots within these regions have been investigated [9]. Sixty-five subjects (35 with DNA-binding domain mutation and 30 with SH2 domain mutation) were evaluated and suggest increased non-immunologic features in the SH2 domain group including high palate, increased interalar distance as well as increased scoliosis in a younger patient subset. They also showed a possible association with increased mortality risk secondary to infection as 6 of the 7 infection related deaths had DNA-binding domain mutations (five in the arginine 382 hotspot). Causative organisms were not reported but a trend of increased Varicella-Zoster reactivation was observed [9]. Although these findings are suggestive, this sample size still lacks statistical power which has been true in other studies as well [10,11]. Our patient has a mutation in SH2 domain and does not have the aforementioned non-immunologic findings, but over time they could develop.

### Role in infection

Disseminated histoplasmosis in HIES is rare and not yet reported in young children. We found six case-reports total (5 patients) in the literature of histoplasma infection in HIES. Three had gastrointestinal tract disease (two teenagers and one adult) [12]. One patient had laryngeal histoplasma only [11]. The other case, reported twice, involves an adult male with budding yeast from a tongue ulcer and histoplasma cultured from right middle lobe infiltrate [11,13]. Invasive fungal disease in HIES is typically seen in adults and attributed to lung damage from previous bacterial pneumonias [11]. Our patient developed pneumatoceles following pneumococcal pneumonia that possibly served as a nidus for Histoplasma, which ultimately disseminated. There are reports of other invasive mycotic diseases [14]. These cases suggest increased risk of disseminated fungal infection with endemic mycoses in HIES patients, raising the possibility that STAT3 has a role in intracellular infection.

Fungal pathogens are recognized by C-type lectin receptors (Dectin-1, Dectin-2, macrophage-inducible C-type lectin, mannose receptor), and there are recent reports indicating variants of either these receptors or down-stream signaling molecules will result in defective NFkB function. NFkB normally promotes IL1β, IL-6 and IL-23 cytokine production by macrophages [15]. These macrophage cytokines facilitate STAT3 dependent Th17 cell differentiation. Th17 cells produce cytokines (i.e. IL-17, IL-21, IL-22) crucial for host defense against bacteria and fungi [15]. Thus if STAT3 signaling is defective, as in HIES, Th17 differentiation is suppressed and there is resultant susceptibility to opportunistic infection [15].

HIES and chronic mucocutaneous disease (CMCD) share the clinical similarities of recurrent staphylococcal and candidal skin infections resulting from defective Th17 T cells. IL-17A and IL-17F are members of the IL-17 family that seem to play an important role in prevention of these infections. It has been recently reported that deficits in IL-17 production result in defective salivary activity due to antimicrobial protein impairment suggested to play a major role in initial fungal defense [16]. In addition, IL-22 stimulation results in bolstering the innate response through its production of β-defensins. Recently described autosomal recessive defect in IL-17 receptor A (IL-17RA) and autosomal dominant defect in IL-17F are both associated with increased staphylococcal and candidal infections [17]. In HIES, "cold boils" and recurrent pulmonary infections by *Staphylococcus aureus *or *Haemophilus influenza *are common.

A commonly described opportunistic fungal infection in primary immunodeficiency is *Pneumocystis jiroveci *although it is more often described in T-cell immunodeficiences. *P jiroveci *(formerly P carinii) is an extracellular fungus that has been described in several patients with HIES. Holland et al report seven HIES, HIV-negative patients with *P jiroveci *found in respiratory or pulmonary specimens [18]. Those patients still living responded to appropriate therapy and were not continued on prophylaxis. Whether *P jiroveci *susceptibility in HIES is due to preceding chronic lung disease or immunologic abnormalities is not known. The IL-23-IL-17 cytokine axis is important in the murine model via IL-23 leading to increased production of IL-17 and IL-22 [19,20]. Our patient did have BAL findings of extracellular yeast and thus was treated with three weeks of Bactrim, but he never had a definitive diagnosis of *P jiroveci *pneumonia.

### Scoring systems

As previously mentioned over 30 patients with HIES were used to develop a clinical scoring system in 1999 (NIH-HIES score) [1]. A more recent scoring system (referred to as the HIES STAT3 score) published in February 2010 attempted to define clinical features that were better predicators of genotype [2]. The authors studied over 100 patients with presumed HIES and IgE levels > 1,000 IU/mL to determine the five features that comprise their criteria: pneumonia, newborn rash, pathologic fractures, characteristic face of Job's syndrome and cathedral palate [2]. The terminology used is more consistent with immunodeficiency diagnostic criteria of other more studied diseases.

The newer scoring system also utilizes Th17 cell counts in its diagnostic guidelines. Multiple groups have shown reduced Th17 cell counts expressed as percentage of CD4+ T cells in HIES [2,10,21,22]. This test is not currently commercially available, but has been shown helpful in differentiating between HIES versus atopic dermatitis [22].

Our patient's clinical scoring was recorded over time and increased with sequential calculations. This trend exemplifies how patients accrue symptoms over time making early diagnosis in young children very difficult.

### Confounders

Defects in STAT3 signaling and its pathobiologic implications on immunologic and non-immunologic pathways will not completely manifest upon initial presentation. Development of additional symptoms over time is the expectation in HIES. For this reason, the NIH scoring system has a young age correction since certain parameters (i.e. multiple pneumonias, retention of primary teeth, scoliosis) will not be present until later in life. Young children can often have additional confounding co-morbidities that lead to unnecessary evaluations and delay in diagnosis.

Our patient's symptoms could be attributed to other possible etiologies. He had multiple rib fractures at different stages of healing that were suspicious for non-accidental trauma. With his osteopenia, he was predisposed to increased risk for fractures. Additionally, his multiple inhalant and food allergies could have contributed to eosinophilia and elevated IgE levels. Multiple pneumonias in children with gastroesophageal reflux are common due to aspiration. Our patient had severe reflux that required G-tube and Nissen fundoplication. As he was a late premature infant with a transiently low IgA level, increased risk for infection was expected. Both IgA and IL-17 responses are important in defense against fungi [23]. Moreover, B cell immunoglobulin production and Th17 cells can be primed by Dectin-1 [24], although we have not investigated the Dectin-1 response in our patient.

There are many pediatric diagnoses much more common than HIES that could explain the aspects of our patient's presentation. Therefore, like our patient, young children are more prone to have a delay in diagnosis, whereas older children and adults will have accumulated more hallmark symptoms. The total IgE level can trend down to under 1,000 IU/mL, although values do not correlate well with disease activity or severity. Thus, clinical suspicion, coupled with scoring systems repeated over time can facilitate ultimate diagnosis.

### Management

As with most primary immunodeficiences without a cure, AD-HIES management focuses on preventative measures to limit the number and severity of infections. Our patient will remain on indefinite prophylactic antibiotic and antifungal therapy. Although he initially responded to booster with Prevnar 13, his immunity waned over 9 months to only one protective titer. Institution of prophylactic antibiotics targeted against pyogenic bacteria (namely *Staphylococcus aureus*) is paramount. Second-generation cephalosporins or trimethoprim/sulfamethoxazole are options for antibacterial prophylaxis; however, if lungs have structural damage, antimicrobial coverage is broadened to include Pseudomonas. History of fungal infection requires prophylactic treatment with fluconazole or itraconazole. Aggressive treatment at the earliest sign of infection is necessary to prevent serious illness. As these patients may have only mild symptoms due to impairment of acute phase cytokines (i.e. IL-6), symptoms may not correlate with severity of disease. As previously reported, routine PJP prophylaxis does not appear to be necessary [6]. For patients with poor antibody production, IVIG can be considered. There are several case reports finding benefit in using IVIG for acute infection or prophylaxis, but no controlled studies exist at this time.

In addition to defects in the immune system, there are also non-immunologic disease manifestations that must be managed. Skin care focuses on reduction of bacterial colonization on the skin. Frequent bleach baths followed by moisturizing and adequate control of pruritus are recommended. Optimizing vitamin D and calcium intake is helpful for those patients with osteopenia. A predilection towards aneurysms makes blood pressure control important. Evaluation for retention of primary teeth and scoliosis in childhood is necessary. There should also be monitoring for signs and symptoms of lymphoma due to the increased incidence in AD-HIES. These patients have a chronic disease that can result in generalized or specific pain, depression, anxiety and fatigue. If chronic symptoms are affecting quality of life, then complementary medicine such as acupuncture can be attempted [25].

Hematopoietic stem cell transplant (HSCT) is generally ineffective in type 1 HIES [26,27]. More recently, however, myeloablative allogeneic HSCT proved effective in two unrelated boys with AD-HIES and non-Hodgkin's lymphoma who continue to have normal immune reconstitution and no progression of non-immunologic manifestations at follow-up 10 and 14 years [28].

### Autosomal recessive forms

In addition to the autosomal dominant inheritance patterns of HIES there are less prevalent forms of autosomal recessive inheritance (Table 2). Tyrosine kinase 2 (TYK2) defect was discovered in 2006 and dedicator of cytokinesis 8 protein (DOCK8) defect in 2009 [29,30]. These autosomal recessive forms share some of the immunologic manifestations of AD-HIES but do not have the same non-immunologic findings. TYK2-HIES has been described in one patient and DOCK8-HIES has been described in < 100 patients to date. They both have recurrent cutaneous and pulmonary bacterial and fungal infections, eczema, eosinophilia and elevated IgE. TYK2-HIES does not have development of pneumatoceles and both forms are distinct given their susceptibility to viral infections such as herpes and molluscum contagiosum [31]. Both forms are also prone to allergic rhinitis and food allergies whereas this finding is atypical in AD-HIES. DOCK8-HIES is also associated with susceptibility to Salmonella and Giardia infections as well as development of early onset malignancies and central nervous system vasculitis [31]. These patients have a poor prognosis. Stem cell transplant has been successful in DOCK8-HIES [32-34].

**Table 2:** Clinical and management features of different forms of HIES

Mutation	Inheritance	Cases reported	Clinical distinctive characteristics	Management
**STAT3**	AD	300-400 (in U.S.); 0.5-1 per million	Skeletal and dental abnormalities	Non-immunologic evaluation and treatment, prophylactic antibiotics +/- antifungals
**TYK2**	AR	1-2 (none in U.S.)	No pneumatoceles, recurrent viral infections, mycobacterial infection	Prophylactic antibiotics +/- antifungals +/- antivirals
**DOC8**	AR	50-60	Recurrent viral infections, food allergies/rhinitis, lymphopenia, increased risk of malignancy, CNS vasculitis	Prophylactic antibiotics and antivirals +/- antifungals, IVIG if antibody deficiency, allogeneic hematopoietic stem cell transplantation

Our patient and another patient previously reported by our group [35] were found to have significant inhalant and food sensitivity which is uncommon in AD-HIES. The previously reported case had IgE sensitization proven by skin and serum testing as well as clinical correlation by double-blind, placebo-controlled challenge to cow's milk. In both cases avoidance of milk protein and usage of elemental formula was an effective treatment.

## Conclusions

This case underscores the inherent diagnostic difficulty of HIES in young children with confounding co-morbidities (food and inhalant allergies which are unusual features for STAT3-HIES, transiently low IgA, aspiration pneumonia, etc.). Clinical scoring systems establish threshold scores for HIES suspicion: 40 and 30+IgE > 1,000 IU/ml, in the NIH-HIES and HIES STAT3 systems, respectively [1,2]. Utilizing clinical scoring systems and maintaining an appropriate level of suspicion, clinicians can determine when STAT3 mutation analysis is necessary. Availability of peripheral blood Th17 cell enumeration at a clinical diagnostic laboratory would allow for more expedient diagnosis and help to distinguish HIES from other diseases with elevated IgE, namely atopic dermatitis [22]. Prophylactic therapy with antibacterial and antifungal agents (when indicated) are key points in patient management, along with regular monitoring of vaccine titers and proactive vaccination. This case of STAT3-HIES also highlights the inherent susceptibility of these patients to intracellular organisms endemic to the region (in our case, histoplasmosis). The clinical course and outcome for our patient may be instructive for other physicians managing patients with STAT3-HIES.

## Competing interests

The authors declare that they have no competing interests.

## Authors' contributions

WSR contributed to literature search relevant to STAT3, interpretation of the results, and wrote the first draft of this report. SRA contributed to the diagnostic procedures for disseminated histoplasmosis, formulated treatment and prevention regimen, wrote the relevant section, and critically revised the report. CFM contributed to overall diagnostic procedures and management, and critical revision of the report. JDV carefully assessed HIES scores (Table 1), contributed to literature search, the section pertaining to the Th17 cell and fungal infection pathogenesis aspect. RAS contributed to the critical diagnostic procedures for disseminated histoplasmosis and immune deficiency, formulated treatment regimen, and critically revised the manuscript. EKP contributed to genetic evaluation and interpretation of the STAT3 variant results, and critical manuscript revision. JCW contributed to genetic evaluation, interpretation of the STAT3 variant results, and critical manuscript revision. VN conducted Th17 cell analysis. DBL directed overall diagnostic priorities and management, contributed to literature search and manuscript preparation. All authors read and approved the final manuscript.
